# Assessment of the Tribological Properties of Aluminum Matrix Composites Intended for Cooperation with Piston Rings in Combustion Engines

**DOI:** 10.3390/ma15113806

**Published:** 2022-05-26

**Authors:** Anna Janina Dolata, Jakub Wieczorek, Maciej Dyzia, Michał Starczewski

**Affiliations:** 1 Faculty of Materials Engineering, Silesian University of Technology, Krasińskiego 8 St., 40-019 Katowice, Poland; jakub.wieczorek@polsl.pl (J.W.); maciej.dyzia@polsl.pl (M.D.); 2 ZŁOTECKI Ltd., Żelechlin 2 St., 88-110 Rojewo, Poland; michal.starczewski@zlotecki.pl

**Keywords:** aluminum matrix composites (AlMCs), ceramic reinforcement, silicon carbide, glassy carbon, tribological properties, piston, piston rings

## Abstract

Tribological interactions between the piston groove and ring in combustion engines have a significant influence on mechanical friction losses. Based on the analysis of the distribution of forces acting on the piston, the conditions for the friction tests were selected. The research was carried out on composites reinforced with silicon carbide (SiC_p_), glassy carbon (GC_p_), and a hybrid mixture of particles (SiC_p_ + GC_p_). Tribological tests were carried out under extremely unfavorable dry sliding conditions using a pin-on-block tester. The friction of coefficient and wear values of the matrix alloy, composites, and iron were compared. Profilometry was used to perform quantitative and qualitative analyses of the wear tracks formed on the tested surfaces. The effect of the presence of reinforcing particles on the geometry of working surfaces was also evaluated. The obtained results show that AlSi12CuNiMg/SiC_p_ and AlSi12CuNiMg/SiC_p_ + GC_p_ composites provided satisfactory effects towards stabilizing the friction coefficient and reducing the wear of tested tribological couples. This may provide a new solution dedicated to an important system, which is the piston groove/piston ring in diesel engines.

## 1. Introduction

In the automotive industry and in certain areas of the heavy machine industry, road machinery, power generators, agricultural machinery, and piston engines are still the main power sources. This has caused a constantly growing interest in new construction solutions to increase the efficiency of combustion engines with different purposes. Innovative work is being conducted in a few different related areas. There is a search for effective and efficient new ways of lubrication and effective methods for using the heat generated during engine operation [1]. Increasing the strength and reliability of both the engine and its main components, while reducing fuel consumption and toxic emissions of exhaust gases, is also being pursued [2,3]. Therefore, innovative work is carried out in many different areas, from control automation and new construction solutions to materials engineering. In the field of materials engineering, the main role is played by research and the implementation of metal matrix composites (MMCs). Particularly, composites based on lightweight aluminum alloys (AlMCs) reinforced with hard ceramic particles, mainly oxides (i.e., Al_2_O_3_, SiO_2_, and TiO_2_) or carbides (SiC, TiC, and TiC) show great potential [4,5,6,7,8,9,10]. Due to their unique properties and the ability to tune these properties, AlMCs constitute an interesting group of materials dedicated to the construction of innovative internal combustion engines, as well as gas compressors and reciprocating pumps [11,12,13].

In practice, aluminum piston suppliers use a wide range of optimized alloy compositions that are generally based on silumins (such as Al-7% Si hypoeutectic, Al-12% Si eutectic, and hypereutectic alloys with 18 and 24% Si) [14,15,16,17]. Particle-reinforced aluminum matrix composites (PAIMCs), mainly those with silicon carbide (SiCp), are very promising and may provide an alternative to the currently used un-reinforced Al–Si alloys [18,19,20,21,22]. Compared to matrix alloys, AlSi/SiC_p_ composites are characterized by a higher strength and rigidity—especially at elevated temperatures—more stable thermal coefficients of expansion, and increased hardness and resistance to abrasive wear [23,24,25,26]. Based on the tribological and thermal properties of such composite materials, the author’s works [13,27,28] have focused on the concept of using this kind of material in the piston-rings-cylinder liner (PRC) assembly.

### Forces Acting on the Piston Compression Rings

To ensure the proper application of materials, including composite materials, it is essential for understanding the force distribution on the construction element. During operation, a combustion engine piston is under a complex system of stresses, which has been widely discussed, analyzed, and described by many authors [29,30,31,32,33,34].

**Figure 1:**
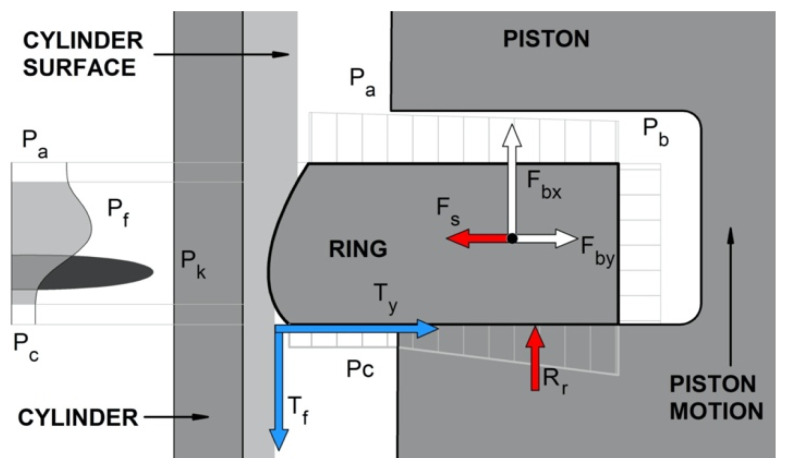
Forces acting on the first piston compression ring, where *P*_a_, *P*_b_, *P*_c_—pressure in volumes above, behind, and below the ring, respectively; *F*_bx_, *F*_By_—ring inertia forces, acting in the axial and radial directions; *F*_s_—ring elastic force; *T*_f_, *T*_y_—ring friction forces acting on the cylinder liner surface and piston groove shelf, respectively; *P*_f_, *P*_k_—oil film pressure and contact pressure with surface irregularities, respectively; and *R*_r_—reaction force of the piston shelf, based on ref. [29].

It should be noted that the main function of piston rings is to seal the combustion chamber against the crankcase. In turn, inter-ring dynamics are highly affected by inter-ring fluid dynamics, and many authors have considered both phenomena together, similar to Selmani et al. [34]. The forces acting on the piston rings were discussed by Niewczas et al. [29]. The authors presented several models that took into account the twists of the rings, the presence of an oil film, and the surface roughness and surface wear of the ring and groove. Similar models were shown by Wolff et al. [30,31,32]. The authors developed a complex model for the piston ring operation, taking into account a model of the gas flow through the labyrinth seal piston-rings-cylinder and a model of the oil flow in the lubrication gap between the ring and cylinder liner [32]. They pointed out that at high temperatures the oil viscosity is very low and consequently the oil film thickness is very thin along the cylinder liner. In this case, it is important to consider the mixed lubrication model and surface roughness of materials. The effect of the top ring geometry profile on the tribological characteristics of a low-displacement diesel engine was analyzed by Furero et al. [33]. The authors observed that the geometric profile of the compression ring modified the friction force caused by surface roughness. The scheme of the forces acting on the compression piston ring, prepared based on ref. [29], is shown in Figure 1. The most general form of the ring sliding surface profile and the partial oil filling of the gap between the ring and cylinder was assumed, similarly to refs. [29,30,31,32,34].

The main forces acting on the piston compression ring (Figure 1) in the axial direction can be characterized as follows:

- *P*_a_—the pressure acting on the upper-surface ring. In the case of the top compression ring, it is often assumed to be the pressure inside the combustion chamber;
- *P*_c_—the pressure from the inter-ring space (below the ring) acting on the lower surface of the ring (the surface protruding beyond the piston groove). In this case, the force is directed upwards and tries to lift the ring from the lower shelf of the piston groove;
- *T*_f_—the friction force acting on the working surface of the ring in contact with the cylinder (always acts opposite to the piston motion). It lifts and pushes the ring towards the lowers the piston groove shelf. This force is affected by the ring elastic force (*F*_s_), the pressure of the ring gases on the cylinder surface, and the quality of lubrication;
- *F*_bx_—the axial component of the inertia force (always directed opposite to the acceleration direction of the piston). In the case of the upper position of the piston, this force tends to pull the ring away from the lower grove shelf. While, in the lower position, it presses the ring to the shelf;
- *R*_r_—the reaction force of the lower piston shelf on the side surface of the ring, directed opposite to the force pressing the ring to the shelf.

**Figure 2:**
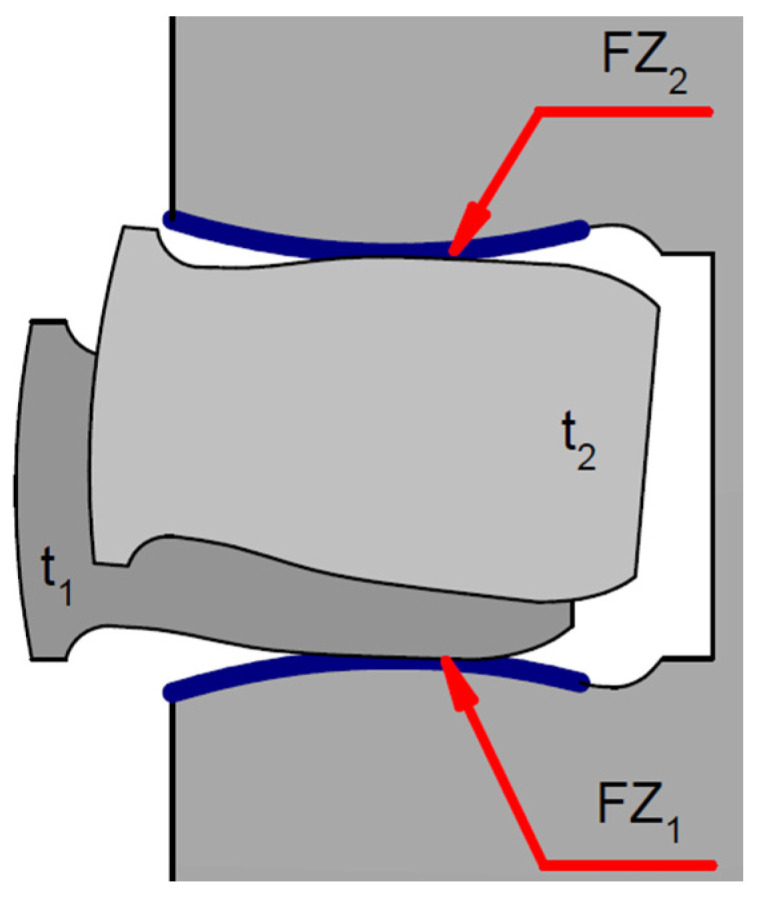
Wear of the piston ring and piston shelf as a result of their cooperating, where: *t*_1_—ring position relative to the piston groove shelf at time *t*_1_; *FZ*_1_—friction zone (ring—lower shelf) at time *t*_1_; *t*_2_—ring position relative to the piston shelf at time *t*_2_; and *FZ*_2_—friction zone (ring—lower shelf) at time *t*_2_.

In the radial direction (Figure 1), the piston ring is affected by the following forces:

- *F*_s_—the force caused by the self-elasticity of the ring, which is dependent on the ring construction;
- *P*_a_—the force created by the working agent pressure, acting radially on the ring. This is the main part of the pressing and sealing forces of the PRC assembly;
- *T*_y_—the friction force between the side surface of the ring and the piston groove. It works during the radial movement of the ring and can be caused by piston torsion inside the cylinder when the movement direction changes or if the cylinder is deformed. This force, depending on the piston position, can lift or press down the ring against the cylinder surface (Figure 2).

As a result of the cooperation of the piston ring with the surface of the piston groove, the shape of the geometric profile of these elements changes (Figure 2). The scale of the wear phenomenon near the piston groove and the piston ring was discussed by Niewczas et al. [29]. The authors estimated that after 300 h of engine operation (which is about 30,000 km during normal operation conditions), the value of the nominal piston ring clearance in the groove doubled.

**Figure 3:**
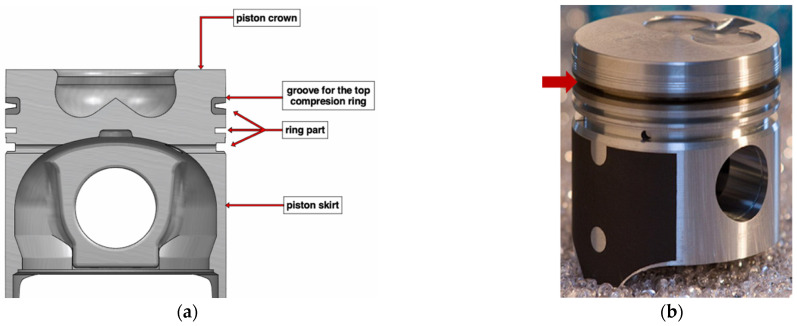
Piston for diesel engines with a cast iron insert: (**a**) Scheme of the piston cross-section divided into key components and (**b**) View of the piston; the arrow marks the area reinforced with a cast iron insert [37].

Due to the above, to increase the wear resistance in highly loaded diesel engines, a cast iron insert is used for the first ring groove (Figure 3). The most important issue for such a solution is the strong connection between the ring carrier and piston material. Therefore, a special Al-Fin casting process should be carried out to prepare the ferrous surface before casting non-ferrous Al alloy pistons [35,36].

Our research focuses on the possibility of using AlMCs as an alternative to the materials that are currently used in the production of pistons, cylinder liners, and cast-iron inserts. Compared with previous works [13,27,28], the tribological properties of composite materials were assessed after cooperation with an iron sealing ring applied in the internal combustion engines (4CT90 produced by Andoria S.A., Andrychów, Poland).

## 2. Materials and Methods

### 2.1. Matrix, Reinforcement, and Composite Materials

**Figure 4:**
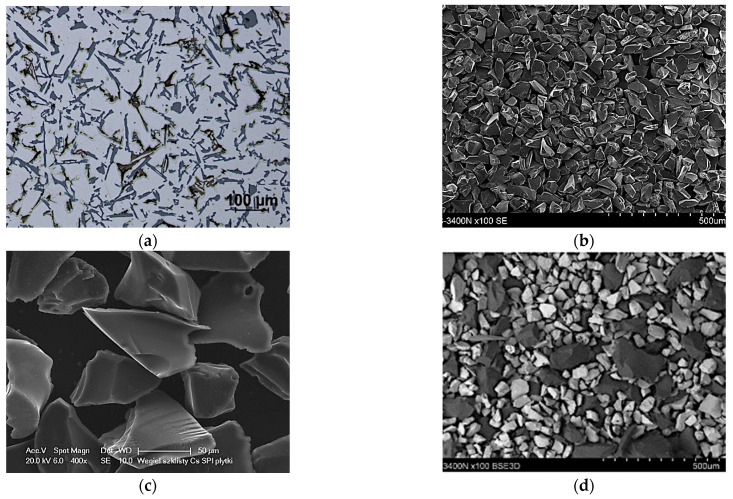
Microstructure of the base AlSi12 matrix alloy and the morphology of ceramic reinforcement: (**a**) AlSi12CuNiMg, LM; (**b**) silicon carbide (SiC_p_), SEM; (**c**) glassy carbon (GC_p_), SEM; and (**d**) SiC_p_ + GC_p_ hybrid powder mixture, SEM.

The tested materials were PAlMCs based on the EN-AC 48000 aluminum alloy (AlSi12CuNiMg) reinforced with SiC_p_, GC_p_ ceramic particles, and their hybrid powder mixture (SiC_p_ + GC_p_). The microstructure of the un-modified AlSi12 matrix alloy and the morphology of reinforcing phases is shown in Figure 4.

**Figure 5:**
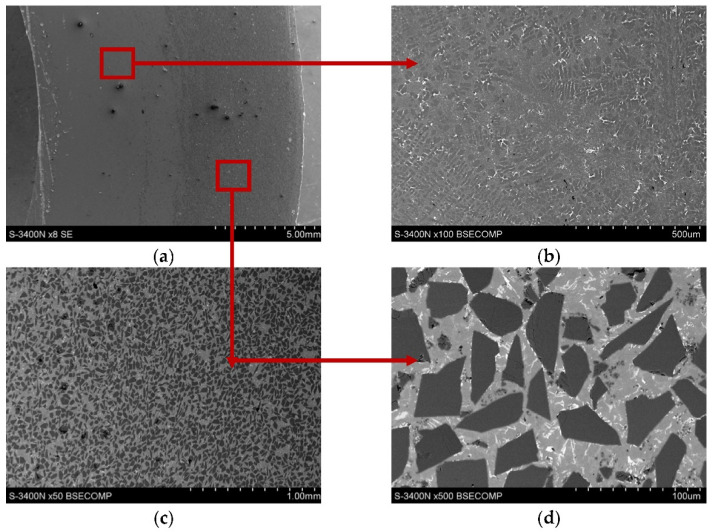
Cross-section perpendicular to the rotational axis of centrifugal cast obtained with AlSi12CuNiMg2/SiC_p_ 10 wt% composite suspension; the red arrows indicates the microstructure of the Al/SiC_p_ composite layer: (**a**) macrostructure with visible outer composite layer, SEM; (**b**) microstructure of modified Al matrix alloy (area without SiC_p_), SEM; and (**c**,**d**) microstructure of SiCp particle-rich region, SEM.

To improve the wetting between metal and ceramic compounds [38], the chemical composition of the eutectic base AlSi12 alloy was modified by adding 1% Mg (Table 1). For this purpose, an AlMg25 master alloy produced by the Institute of Non-Ferrous Metals in Skawina was used. The composite suspension was obtained by a stir-casting method and formed into the sleeves using a centrifugal casting process. The technological procedures have been described in previously published papers [27,28,39,40,41]. The microstructure of AlSi12CuNiMg2/SiC_p_ 10 wt% composite is shown in Figure 5.

### 2.2. Tribological Studies and Profilometry Analyses

**Figure 6:**
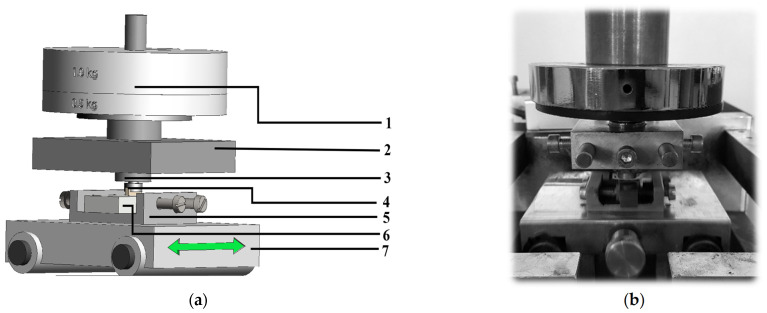
The experimental pin-on-block set-up used in tribological tests: (**a**) scheme of the stand, where: 1—Load, 2—strain gauge holder, 3—sample holder (pin), 4—cast iron pin, 5—sample holder (block), 6—composite sample, and 7—movable plate; and (**b**) view of the stand.

The tests of the friction coefficient (COF) and wear of the materials were conducted at room temperature (*T* = 20 °C) under technically dry friction conditions using a pin-on-block tribo-tester (Figure 6, Table 2). The scope of work was as follows:

- Preparation of samples through cutting the composite sleeves into discs ~5 mm thick;
- Preparation of counter-pins by cutting out a fragment of the sealing ring and attaching it to the holder;
- Tribological tests on a block-pin tester in dry friction conditions;
- Measurement of the friction coefficient and profilometry analysis of the wear traces.

During tribological tests, the friction load was measured continuously by using a strain gauge connected with an analog-digital converter and recorder. Using the registered values, the coefficient of friction (μ) was calculated as a function of sliding distance based on the author’s software. The software is a dedicated statistical calculator that was designed to analyze a large number of measurement points (time and force) recorded during tribological tests. It averages sector data and presents them in the form of graphs of friction distance versus COF. The designation of the friction pairs used in tribological tests is presented in Table 3.

The analysis of the friction area of composites and cast-iron pins was conducted using profilometry measurements using a MicroProf 3000, FRT optical profilometer with an WCL3000 aberration head, (FRT GmbH, München, Germany) [28]. It is a non-contact measurement, so it completely eliminates the possibility of deforming the friction trace during the test. A study of the wear trace geometry was conducted immediately after the friction process. Only an ethanol solution was used to clean the surface of the tested samples. The surface geometry was evaluated using the following parameters: measurement area of 8 × 8 mm^2^; resolution of measurement points of 1600 × 1000; and image acquisition and data processing using MARK III software (FRT GmbH, München, Germany). Based on three-dimensional (3D) images, basic surface characteristics such as depth of the wear trace (*Sa*), roughness (*Ra*), and volume loss of analyzed materials were assessed.

## 3. Results and Discussion

### 3.1. Coeficient of Friction

**Figure 7:**
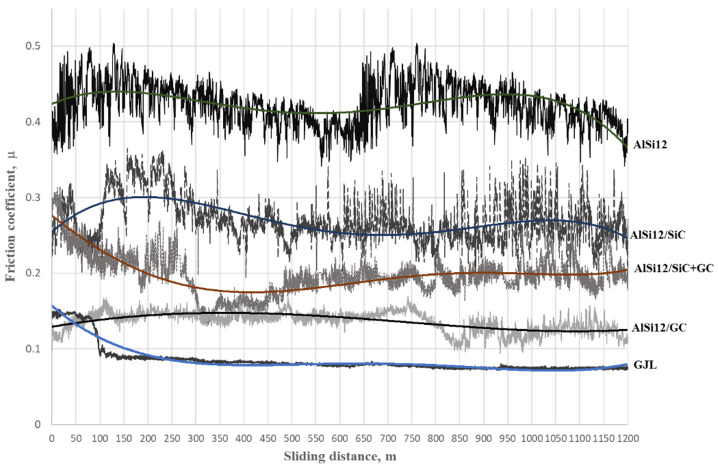
Friction coefficient (μ) versus sliding distance for the tested materials; the change of the trend for the tested materials was marked with continuous lines.

The coefficient of friction (COF) as a function of the sliding distance for the tested materials is shown in Figure 7. In the case of the unreinforced matrix alloy (AlSi12), changes in the COF were unstable, with an average value of more than 0.4. As can be seen, the value of the COF during the entire sliding distance changed from μ = 0.35 to μ = 0.5. Such a high variation (equal to 0.15) is unacceptable. Such sudden changes in the COF are characteristic of AlSi alloys, but the obtained result provides a reference point for assessing the influence of reinforcing particles on the tribological properties of the tested composites.

As can be seen, the changes in the COF were significantly different for the cast iron–cast iron tribological pair (Table 3). In this case, the value of the COF after a short run-in period (100 m) stabilized at μ = 0.07 (Figure 7). Over the entire sliding distance, this value did not change, giving a very stable tribological cooperation.

Due to the use of silicon carbide as reinforcing particles (SiC_p_), the effect of decreasing the value of the friction coefficient was obtained compared to the un-reinforced AlSi12 matrix alloy. However, in both cases, a similar unstable change in the COF was observed (Figure 7). During the sliding distance, the COF of the AlSi12/SiC_p_ composite increased and decreased in the range of μ = 0.22 to μ = 0.32. In turn, the use of the hybrid reinforcement mixture (SiC_p_ + GC_p_) decreased the COF (μ = 0.2) and partially stabilized it, particularly after the brake-in period (500 m). The stabilization effect of the COF was significantly visible in the case of the AlSi12/GC_p_ composite. As can be seen (black line in Figure 7), the COF of the AlSi12/GC_p_ was significantly lower than the other tested composites (μ = 0.12). In the final stage of friction, the difference between the maximum and minimum values did not exceed 0.02 and was comparable to the values measured for iron materials.

### 3.2. Wear

**Figure 8:**
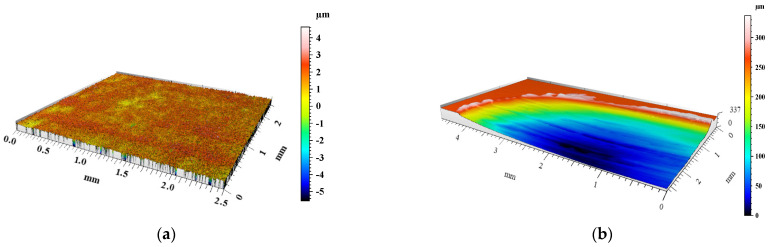
View of the AlSi12 matrix surface geometry: (**a**) before and (**b**) after dry friction test.

**Figure 9:**
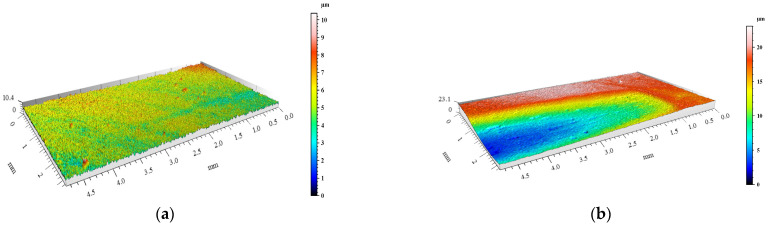
View of GJL cast-iron surface geometry: (**a**) before and (**b**) after dry friction test.

**Figure 10:**
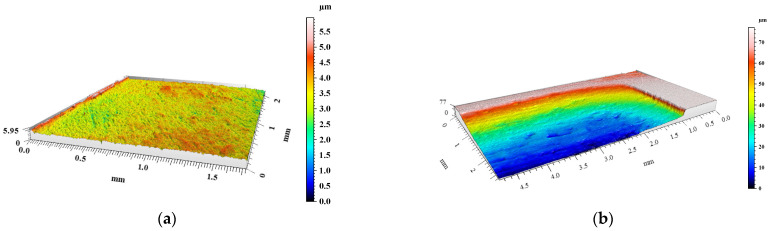
View of AlSi12/SiC_p_ composite surface geometry: (**a**) before and (**b**) after dry friction test.

**Figure 11:**
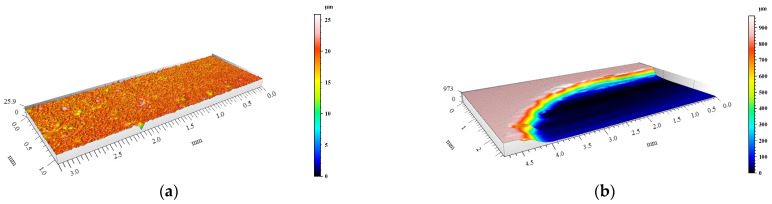
View of AlSi12/GC_p_ composite surface geometry: (**a**) before and (**b**) after dry friction test.

**Figure 12:**
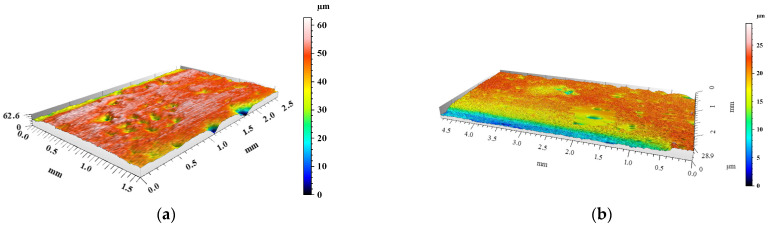
View of AlSi12/SiC_p_ + GC_p_ hybrid composite surface geometry: (**a**) before and (**b**) after dry friction test.

The degree of surface wear under dry friction conditions was determined using the profilometry measurements. The volume loss of materials and the changes in surface geometry resulting from friction were evaluated. 3D images of the created wear tracks compared with the initial surfaces before friction of the materials are shown in Figure 8, Figure 9, Figure 10, Figure 11 and Figure 12. The analysis of 3D images allowed for quantitative and qualitative assessments and an indication of the wear mechanism.

**Figure 13:**
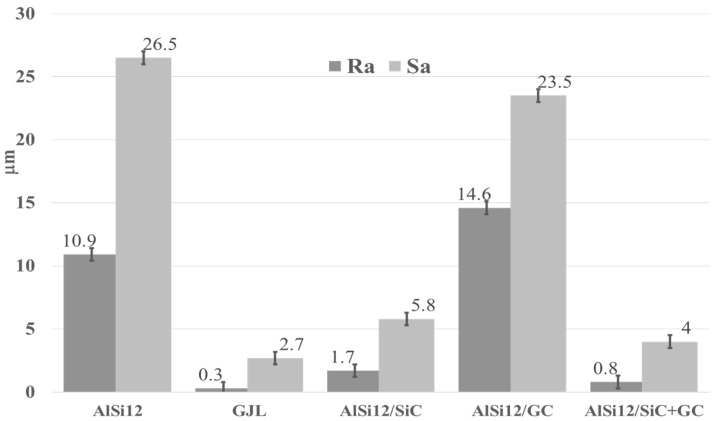
Roughness parameters value (*Ra*) and (*Sa*) of the wear track for all tested materials.

**Figure 14:**
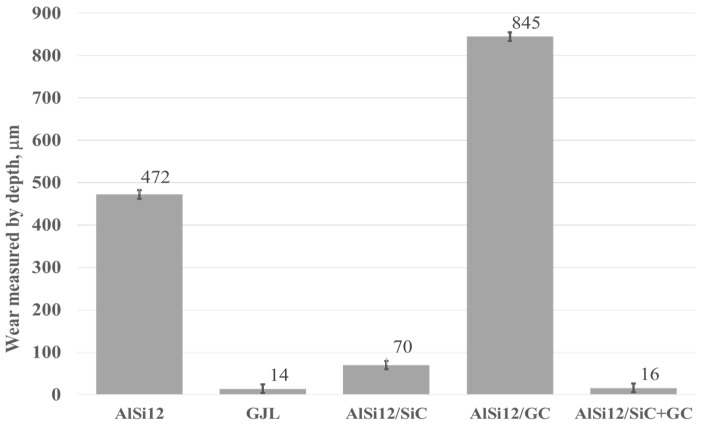
Depth of the wear track calculated based on 100 profiles.

Based on the 3D images (Figure 8, Figure 9, Figure 10, Figure 11 and Figure 12), it can be concluded that all tested materials showed abrasive wear. However, there were significant differences in its intensity that can be compared due to the selected parameters. To evaluate the changes in surface geometry due to friction, the basic roughness parameters were used, including the arithmetical mean roughness value (*Ra*)—calculated as the arithmetical mean of the absolute values of the profile deviations from the mean line of the roughness profile, and (*Sa*)—arithmetical mean surface height (Figure 13), as well as the average depth of the wear trace (Figure 14).

Comparing the obtained images of the surface geometry after friction and the roughness parameters, it can be seen that the most intense wear occurred in the un-reinforced AlSi12 matrix alloy (Figure 8 and Figure 13) and in the composite reinforced with glassy carbon particles (Figure 11 and Figure 13). In turn, the composite containing silicon carbide particles (Figure 10 and Figure 13) and the composite with a hybrid mixture of SiC_p_ and GC_p_ (Figure 12 and Figure 13) were characterized by significantly less abrasive wear.

**Figure 15:**
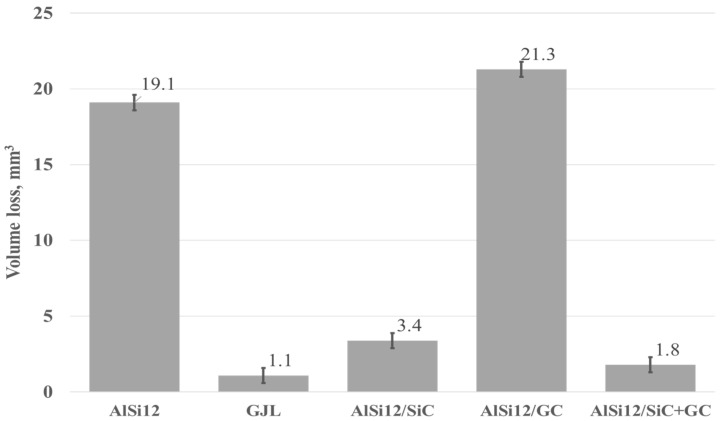
Volume loss of the tested materials calculated based on the wear track created under dry friction conditions.

Based on profilometry measurements and using our software capabilities, the volume loss of the materials was calculated. Then, the volume loss was used to compare the wear resistance of the tested materials. The results in the form of a graph are shown in Figure 15.

**Figure 16:**
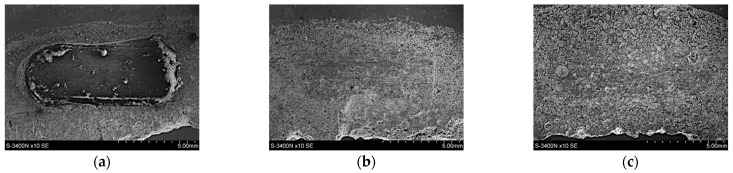
SEM images of the wear tracks on the composite surface: (**a**) AlSi12/GCp; (**b**) AlSi12/SiC_p_; and (**c**) AlSi12/SiC_p_ + GC_p_ hybrid composite: (**a**,**b**) LM; (**c**) SEM.

As can be seen (Figure 15), the highest volume loss, and thus the lowest wear resistance under dry friction conditions, was recorded for the composite reinforced with glassy carbon particles (21.3 mm^3^). Relatively high-volume losses also occurred for the un-reinforced AlSi12 matrix alloy (19.1 mm^3^). In turn, the lowest volume losses, and therefore the high resistance to abrasive wear, was noted for cast iron (1.1 mm^3^). Whereas, among composite materials, the SiC_p_ + CG_p_ hybrid reinforcement was the most advantageous (1.8 mm^3^). In this case, the silicon carbide particles improved the abrasion resistance, while the addition of glassy carbon played an important role as a lubricant for the cooperating surfaces. The SEM images of the wear tracks on the composites surface are shown in Figure 16.

**Figure 17:**
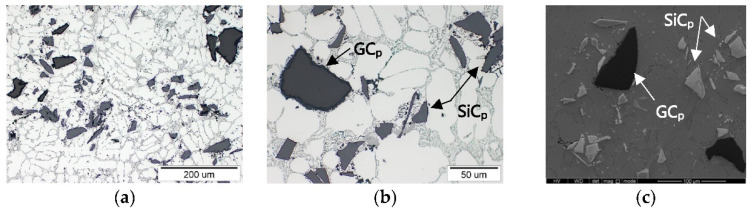
Microstructure of the AlSi12/SiC_p_ + GC_p_ hybrid composite: (**a**) and (**b**) LM; (**c**) SEM.

The obtained results can be related to the specific microstructure of the AlSi12/SiC_p_ + GC_p_ hybrid composite (Figure 17) and to the synergistic strengthening and self-lubricating effect. In this case, two parallel phenomena can be identified in the wear process. The first is the interaction of hard SiC_p_ particles with a cast-iron friction partner. As a result, SiC particles were periodically removed from the matrix alloy, which caused significant wear to the cast iron. The second phenomenon concerns the wear of GC_p_ particles, which were pulled from the matrix and crushed during the friction process, creating a solid sliding layer on the friction surface, which reduced the COF.

As can be seen (Figure 17), in the AlSi12/SiC_p_ + CG_p_ hybrid composite microstructure, larger glassy carbon particles were surrounded by much smaller SiC_p_ particles, which increased the hardness, provided load transfer through the surface, and wear resistance for the AlSi12 matrix alloy. In turn, the GC_p_ particles play an important role as a lubricant for both the working surfaces of the matrix and the SiC_p_ particles themselves. Similar results have been described by Myalski and Sleziona [42]. They found that the high hardness of glassy carbon particles (comparable to SiC particles) did not cause major damage to the cast iron pin that served as the friction partner to the composite material. The authors explained these results by the fact that mainly glassy carbon particles with a low COF participated in the friction process. Furthermore, in our previous research [13,28], it was shown that the presence of glassy carbon, both in the form of particles and spheres in various types of Al/SiC_p_ composites, not only decreased and stabilized the COF but also reduced the wear of the friction partner in tribological couples.

**Figure 18:**
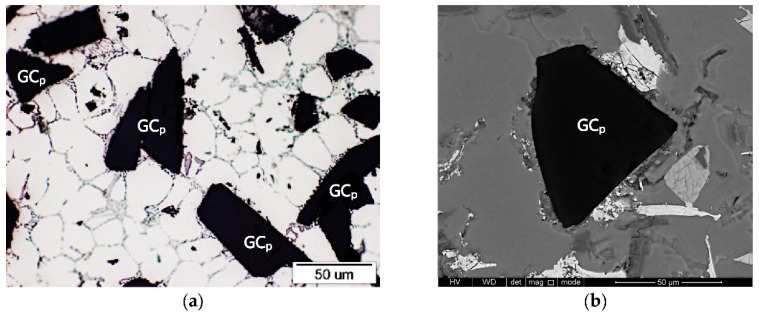
Microstructure of AlSi12/GC_p_ composite: (**a**) LM; (**b**) SEM.

In turn, the presented results confirmed that the presence of single glassy carbon particles inside the aluminum matrix alloy (AlSi12/GC_p_ composite, Figure 18) has a much lower effect on the wear resistance. However, it helped reduce and stabilize the COF.

## 4. Conclusions

The conducted tribological investigation under dry friction conditions allowed for an evaluation of the behavior of materials in extremely unfavorable working conditions.

The obtained results and their analysis clearly indicate that the highest COF was recorded for the un-reinforced matrix, and the average value was µ = 0.42. In this case, a high-volume loss of the material was also observed, at the level of 19 mm^3^. It was shown that introducing reinforcement particles into the AlSi12 matrix alloy significantly changed the friction conditions. However, the glassy carbon particles (GC_p_) decreased the COF (average µ = 0.12) but did not increase the wear resistance of the material. In this case, the volume loss and depth of the friction trace were the highest among all tested materials (21.3 mm^3^ and 0.85 mm, respectively). In turn, the SiC_p_ reinforcement decreased the COF to µ = 0.26 and also significantly reduced the material wear. The wear depth was almost seven times smaller than that of the un-reinforced matrix alloy. The material that gave the most beneficial effect, a low COF (µ = 0.18), and increased the wear resistance (a volume loss of 1.8 mm^3^) was the use of a hybrid reinforcement (SiC_p_ + GC_p_).

The results described here demonstrate that AlSi12/SiC_p_ and AlSi12/SiC_p_ + GC_p_ composites showed significantly better tribological properties than the commercial AlSi12CuNiMg alloy and could be applied to a P-R-C assembly. The results also provide an introduction to further research related to the development of new materials and technological concepts aimed at shaping a local AlSi/SiC_p_ insert in the groove of the top compression rings intended for compression ignition engine pistons.

## Figures and Tables

**Table 1:** Chemical composition ^1^ of EN AC 48000 (AlSi12CuNiMg) commercial alloy * and modified by adding 1% Mg **.

Alloy Composition	Si	Mg	Cu	Ni	Fe	Mn	Ti	Al
AlSi12CuNiMg *	11.98	1.04	0.97	0.89	0.48	0.19	0.05	Bal.
AlSi12CuNiMg2 **	11.48	2.07	2.07	0.85	0.47	0.17	0.05	Bal.

^1^ Alloy composition tested by mass spectrometry (Foundry Master, Oxford Instruments—WAS, Abingdon, UK).

**Table 2:** The parameters used in tribological tests.

Sliding Speed [m/s]	Normal Load [N]	Distance [m]	Temperature [°C]	Unit Pressure [MPa]
0.7	15	1200	20	0.8

**Table 3:** Designation of samples and friction pairs used in tribological tests.

Material Designation	Reinforcing Particles	Volume of Particles [wt%]	Average Particle Diameter [mµ]	Friction Counter Pin
AlSi12	-	-	-	GJL
AlSi12/SiC	SiC_p_	10	58	GJL
AlSi12/GC	GC_p_	10	80	GJL
AlSi12/SiC + GC	SiC_p_ + GC_p_	7 + 3	58 and 80, respectively.	GJL
GJL	-	-	-	GJL

## Data Availability

Not applicable.
